# RNA-Mediated Control in *Listeria monocytogenes*: Insights Into Regulatory Mechanisms and Roles in Metabolism and Virulence

**DOI:** 10.3389/fmicb.2021.622829

**Published:** 2021-04-14

**Authors:** Agata Krawczyk-Balska, Magdalena Ładziak, Michał Burmistrz, Katarzyna Ścibek, Birgitte H. Kallipolitis

**Affiliations:** ^1^Department of Molecular Microbiology, Biological and Chemical Research Centre, Faculty of Biology, University of Warsaw, Warsaw, Poland; ^2^Department of Biochemistry and Molecular Biology, University of Southern Denmark, Odense, Denmark

**Keywords:** non-coding RNAs, post-transcriptional regulation, metabolism, virulence, *Listeria monocytogenes*

## Abstract

*Listeria monocytogenes* is an intracellular pathogen that is well known for its adaptability to life in a broad spectrum of different niches. RNA-mediated regulatory mechanisms in *L. monocytogenes* play important roles in successful adaptation providing fast and versatile responses to a changing environment. Recent findings indicate that non-coding RNAs (ncRNAs) regulate a variety of processes in this bacterium, such as environmental sensing, metabolism and virulence, as well as immune responses in eukaryotic cells. In this review, the current knowledge on RNA-mediated regulation in *L. monocytogenes* is presented, with special focus on the roles and mechanisms underlying modulation of metabolism and virulence. Collectively, these findings point to ncRNAs as important gene regulatory elements in *L. monocytogenes*, both outside and inside an infected host. However, the involvement of regulatory ncRNAs in bacterial physiology and virulence is still underestimated and probably will be better assessed in the coming years, especially in relation to discovering the regulatory functions of 5′ and 3′ untranslated regions and excludons, and by exploring the role of ncRNAs in interaction with both bacterial and host proteins.

## Introduction

*Listeria monocytogenes* is an intracellular, Gram-positive pathogen, responsible for foodborne infections called listerioses in humans and different animal species. This bacterium is well known for its adaptability to life in a broad spectrum of different niches, ranging from soil or wastewater to the cytoplasm of infected mammalian cells. *L. monocytogenes* is widely distributed in the environment owing to its ability to survive in different stress conditions, including pH variations, low temperature and high salt concentration (Ferreira et al., 2014). Infection with *L. monocytogenes* starts with the ingestion of contaminated food. In the intestine, *L. monocytogenes* invades epithelial cells as a result of the interaction of bacterial surface proteins with appropriate eukaryotic receptors. After crossing the intestinal barrier, *L. monocytogenes* enters into macrophage cells and is transported via blood to the liver and spleen. When the host’s cell-mediated response is impaired, *L. monocytogenes* multiplies in these organs and subsequently spreads through the blood to different organs, often crossing placental and blood-brain barriers, leading to septicemia, meningitis and miscarriage in the case of pregnant women (McLauchlin et al., 2004; Liu et al., 2007). *L. monocytogenes* has the ability to invade the host’s cells, multiply inside them and spread from cell to cell owing to tightly regulated expression of genes encoding virulence factors (Cossart, 2011).

The regulation of gene expression has a pivotal role in the virulence of *L. monocytogenes* and the ability of this bacterium to survive in different stress conditions. Proper changes in gene expression programs are indispensable in allowing saprophytic growth, stress response and resistance to extreme conditions, or triggering virulence properties. Numerous studies have documented the importance of protein regulators in the coordination of the infection process. The master coordinator of transcription of the virulence genes of *L. monocytogenes* is transcriptional regulator PrfA (positive regulatory factor A), which belongs to the superfamily of cyclic AMP receptor proteins (Crp) (Renzoni et al., 1999; Reniere et al., 2016). Other regulators of expression of virulence and virulence-associated genes are the alternative sigma factor Sigma B (Dorey et al., 2019), two-component signal transduction systems CesRK, LisRK, and VirRS, as well as the nutrient-responsive regulator CodY (Cotter et al., 1999; Kallipolitis et al., 2003; Mandin et al., 2005; Lobel et al., 2015). While knowledge about protein-mediated control of *L. monocytogenes* gene expression to environmental changes has been acquired over many decades, recent studies have shown that pathogenesis and stress adaptation of this bacterium are also regulated post-transcriptionally by ncRNA molecules. Generally, ncRNAs can be divided into five main categories. The first category contains small regulatory RNAs encoded *in trans* relative to the genes they regulate (*trans* ncRNAs). Some *trans* ncRNAs mainly act through interactions with proteins, whereas others control gene expression through base pairing with RNA transcripts (Storz et al., 2011). The base pairing *trans* ncRNAs affect the translation and/or stability of mRNAs originating from different sites in the genome. They show incomplete complementarity with their targets and thus can interact with multiple mRNAs. The interaction of *trans* ncRNAs with target mRNAs in bacteria is often mediated by the RNA chaperone Hfq (Christiansen et al., 2006). The second category contains *cis*-acting regulatory RNAs encoded from the 5′ regions of the genes they regulate (*cis* ncRNAs). They fold into two alternative RNA structures that terminate or antiterminate transcription of downstream genes. The rearrangements of *cis* ncRNA structures are coupled with translation of small ORFs within their sequences. The third category comprises antisense RNAs (asRNAs), including long antisense RNAs (lasRNAs). These molecules are encoded on the opposite strand relative to the genes they regulate, which makes them perfectly complementary to the target mRNA. Hybridization of asRNAs to target mRNAs often affects their stability and/or translational activity (Waters and Storz, 2009; Wurtzel et al., 2012). The recently discovered excludon corresponds to a genomic locus encoding a lasRNA (Wurtzel et al., 2012). The transcription of an excludon inhibits expression of the gene encoded on the opposite strand and also ensures expression of the downstream operon. The fourth category, viewed by some as the simplest form of RNA regulatory elements, are *cis-*encoded and *cis*-acting molecules, which undergo conformational changes upon binding a specific ligand (riboswitches) or in response to temperature change (thermosensors). Bacterial riboswitches and thermosensors are located mainly in the 5′ untranslated regions (UTRs) and less frequently in the 3′ UTRs of the genes that they control (Waters and Storz, 2009). Conformational changes in riboswitches and thermosensors located in 5′ UTR regions lead to premature transcription termination, arrest of translation initiation or both (Waters and Storz, 2009). Finally, the fifth category is comprised of 5′ and 3′ UTRs, whose regulatory mechanism relies on base pairing with other RNA transcripts.

While this general classification of ncRNAs is widely accepted and very useful due to its simplicity, mounting evidence suggests that ncRNAs are versatile regulators which can act by more than just a single mechanism as will be shown in this review.

**FIGURE 1:**
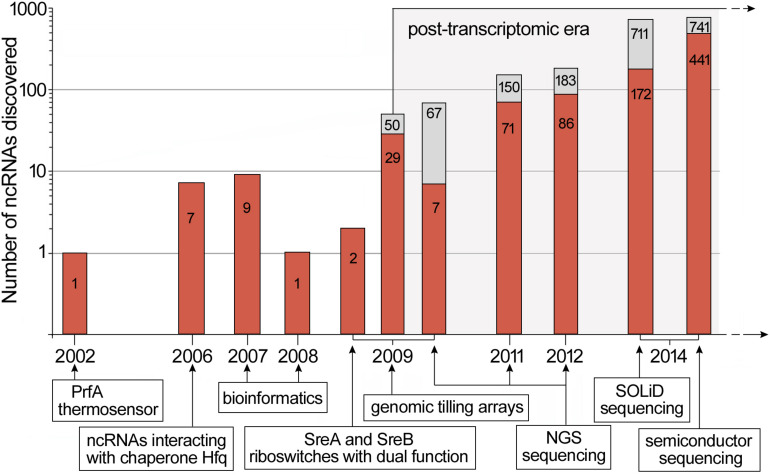
Discovery timeline of ncRNAs in *L. monocytogenes.* New ncRNAs discovered are marked in red while the total number of ncRNAs discovered is indicated in gray. For high-throughput RNA analysis, the data represents putative riboregulatory candidates. Below the timeline, key scientific breakthroughs and novel methods applied in ncRNAs discovery are shown. The timeline is based on data presented in Johansson et al. (2002); Christiansen et al. (2006), Mandin et al. (2007); Nielsen et al. (2008), Loh et al. (2009); Oliver et al. (2009), Toledo-Arana et al. (2009); Mraheil et al. (2011), Wurtzel et al. (2012); Behrens et al. (2014), and Wehner et al. (2014).

Research devoted *sensu stricto* to riboregulation in *L. monocytogenes* began in 2002. At that time, it was discovered that the 5′ UTR of *prfA* switches between a structure active at high temperatures and inactive at low ones. This mechanism is driven by a thermosensor, which regulates the expression of *prfA*, thereby controlling the virulence properties of *L. monocytogenes* (Johansson et al., 2002). Over the next several years, other ncRNAs of *L. monocytogenes* were discovered. The first identified and characterized small ncRNAs of *L. monocytogenes* were LhrA, LhrB and LhrC1-5 interacting with chaperone Hfq (Christiansen et al., 2006). Shortly after, further ncRNAs, i.e., RliA, RliB, RliC, RliD, RliE, RliF, RliG, RliH, RliI, and SbrA were identified using classical methods of molecular biology and bioinformatics (Mandin et al., 2007; Nielsen et al., 2008). During this time, the SreA and SreB riboswitches, which can act as *trans* ncRNAs to inhibit translation of *prfA* mRNA, were also discovered (Loh et al., 2009). The biggest scientific breakthrough in the discovery of riboregulatory elements in *L. monocytogenes* took place in 2009, when Toledo-Arana and coworkers presented the first study of the whole transcriptome of this bacterium. From that moment on, an enormous number of new ncRNAs was discovered, and in a few cases, their function and mechanisms of action was revealed. In this research, genomic tilling arrays was applied to compare the whole transcriptome of *L. monocytogenes* during growth in different physiologically relevant conditions including infection-relevant ones, i.e., whole human blood and the intestinal lumen of mice. Investigation of the transcriptome changes allowed understanding of the switching of *L. monocytogenes* from saphrophytism to virulence. These studies led to the identification of 50 ncRNAs, of which 29 were novel ncRNAs with sizes from 77 to 534 nucleotides (nt), including seven asRNAs. Furthermore, comprehensive information about changes in the expression of ncRNAs in different conditions was provided (Toledo-Arana et al., 2009). In the same year another study was performed by Oliver and coworkers, who applied a high-throughput RNA sequencing method with Illumina Genome Analyzer. This study allowed the identification of 67 *L. monocytogenes* ncRNAs expressed in stationary phase of growth, with 60 molecules being previously described (Oliver et al., 2009). Another NGS (next generation sequencing) method, i.e., 454 pyrosequencing was used by Mraheil et al. (2011), in which sequencing of small RNA (below 500 nt) isolated from bacteria growing inside infected macrophages was carried out. This study led to the identification of 150 ncRNAs, whereof almost half had not been previously described (Mraheil et al., 2011). In the following year, NGS was applied to compare the transcriptomes of pathogenic *L. monocytogenes* with non-pathogenic *Listeria innocua* under various growth conditions (Wurtzel et al., 2012). The results of this study revealed the presence of 113 ncRNAs and 70 asRNAs in *L. monocytogenes*, of which 33 ncRNAs and 53 asRNAs had not been previously identified. This research also led to the identification of new lasRNAs that can act as asRNAs and mRNAs. Such a dual function for a lasRNA transcript was first described in *L. monocytogenes* for the lasRNA regulating flagellum biosynthesis (Toledo-Arana et al., 2009). This type of lasRNA was named an excludon. Another study that used a high-throughput SOLiD sequencing platform led to the discovery of 172 as yet undescribed ncRNAs candidates isolated from intracellularly and extracellularly growing *L. monocytogenes* (Behrens et al., 2014). This method led to the identification of nine new asRNAs and additionally revealed that four asRNAs are potentially longer than previously thought and could form lasRNAs (Behrens et al., 2014). In another whole transcriptomic study of *L. monocytogenes* under intracellular and extracellular growth conditions, a semiconductor sequencing technology and bioinformatic analysis pipeline was applied to identify 741 putative ncRNAs in *L. monocytogenes*, 441 of which had never been described before. One of the newly identified lasRNAs was a very long transcript of about 5,400 nt, fully complementary to a region from *lmo2677* up to *lmo2680* and partially to *kdpB* (Wehner et al., 2014). The described progress in the discovery of ncRNAs in *L. monocytogenes* is shown in Figure 1. Altogether, a huge number of candidates for riboregulatory elements in *L. monocytogenes* have been identified throughout the last decade, and their number has been increasing in line with the progress in sequencing technology. Although the number of potential regulatory RNAs identified varies in different works, it is assumed that *L. monocytogenes* possesses more than 55 riboswitches, 100 asRNAs and 150 putative *trans* and *cis* ncRNAs (Schultze et al., 2014; Lebreton and Cossart, 2017). However, in spite of the rapid increase in the number of newly identified regulatory RNAs, their function and mechanism of action is poorly understood. Interestingly, recent studies have shown that ncRNAs are secreted by *L. monocytogenes* into the cytoplasm of infected cells where they modulate the innate immune response through interaction with the RNA sensor RIG-I (Frantz et al., 2019). Therefore, these molecules besides being potent regulators of gene expression, could also play a role as virulence effectors of *L. monocytogenes*. Furthermore, except for the classic riboregulatory elements, recent research has revealed that canonical mRNA can also be involved in regulatory base-pairing interactions extending riboregulatory mechanisms in *L. monocytogenes* beyond non-coding elements (Ignatov et al., 2020; Peterson et al., 2020). In this review, we focus on the riboregulators of *L. monocytogenes* which, besides being consistently identified in high-throughput studies, have been characterized in low-throughput analyses, providing summarized data on their physiological role and, when available, mechanism of action. The detailed characteristics of these riboregulators are presented in Table 1.

**TABLE 1:**
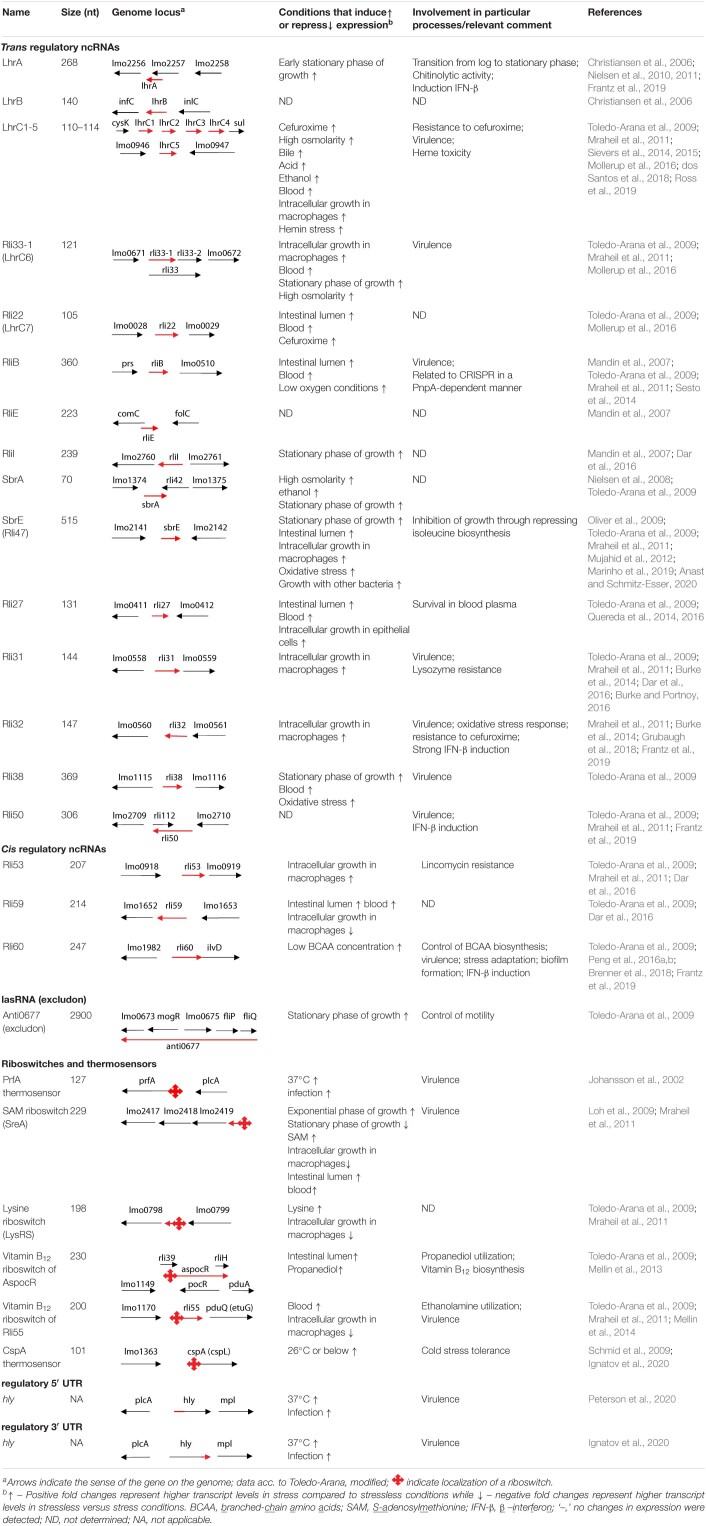
Characteristics of non-coding RNAs of *L. monocytogenes.*

## *Trans* Regulatory ncRNAs

### LhrA

LhrA was identified as an ncRNA interacting with the chaperone Hfq of *L. monocytogenes*. The LhrA transcript is known to be present throughout the growth phase, reaching a maximum level at the onset of the stationary phase, which suggests a role for LhrA during transition from exponential to stationary phase of growth (Christiansen et al., 2006). Notably, the stability of LhrA is highly dependent on the presence of the Hfq chaperone (Christiansen et al., 2006). LhrA is highly conserved among *Listeria* species, and it seems to appear exclusively in the genus *Listeria*. *In silico* studies predicted the secondary structure of LhrA to contain four stem-loops and a well preserved single stranded, 21 nt long motif, which is responsible for base pairing between this ncRNA and its targets. The first identified target for LhrA was *lmo0850*, which encodes a protein of unknown function. The regulation is dependent on Hfq, which facilitates base pairing between LhrA and a region upstream from the start codon of *lmo0850* mRNA, leading to inhibition of translation followed by a decrease in mRNA stability (Nielsen et al., 2010). Further transcriptomic microarray-based studies showed that inactivation of *lhrA* influences the expression level of more than 300 genes in *L. monocytogenes*. Additionally, this study allowed for the identification of two other genes directly regulated by LhrA, i.e., *lmo0302* and *chiA*, encoding a hypothetic protein and a chitinase, respectively. Similarly to regulation of *lmo0850*, these genes are downregulated by LhrA at the posttranscriptional level and the regulation is Hfq-dependent (Nielsen et al., 2011). So far, the dependency on Hfq for efficient binding of LhrA to its targets represents a unique example of Hfq chaperone involvement in ncRNA-mRNA interaction and posttranscriptional regulation in Gram-positive bacteria. A recent study revealed that transfection of eukaryotic cells with LhrA triggers moderate IFN-β induction suggesting the involvement of LhrA in modulating the immune response during infection of the host organism (Frantz et al., 2019).

### LhrC Family (LhrC1-5, Rli33-1, and Rli22)

The LhrC is a multicopy ncRNA family, which comprises seven homologous ncRNAs, ranging from 105 to 121 nt in length. Notably, this ncRNA family holds the highest number of siblings reported so far. The first discovered members of this family were LhrC1-5 owing to their ability to interact with Hfq (Christiansen et al., 2006). LhrC1-5 arises from two different locations within the *L. monocytogenes* genome, i.e., *lhrC1-4* located between genes *cysK* and *sul*, while *lhrC5* resides between *lmo0946* and *lmo0947* (Table 1). Deletion of *lhrC1-5* results in increased susceptibility of *L. monocytogenes* to the β-lactam antibiotic cefuroxime (Sievers et al., 2014), leads to decreased survival of *L. monocytogenes* in macrophage cells (Sievers et al., 2015), and impairs the adaptation of *L. monocytogenes* to excess of heme (dos Santos et al., 2018). While initially five LhrCs were discovered, further studies led to expanding the LhrC family to seven members based on the discovery of two additional ncRNAs, namely Rli22 and Rli33-1, that are structurally and functionally related to LhrC1-5, although they do not possess the ability to interact with the Hfq (Mollerup et al., 2016). Rli22 is encoded from the intergenic region of *lmo0028* and *lmo0029*, and Rli33-1 was initially identified as part of a larger transcript, designated Rli33, encoded from the intergenic region of *lmo0671* and *lmo0672* (Toledo-Arana et al., 2009). A more recent study identified two individual ncRNAs, Rli33-1 and Rli33-2, which suggests either the presence of an internal transcription start site (TSS) or an unknown RNA processing mechanism of Rli33 (Mraheil et al., 2011). Deletion of the *rli33-1* gene resulted in decreased survival of *L. monocytogenes* in macrophage cells and led to attenuation of *L. monocytogenes* virulence in murine and insect models of infection (Mraheil et al., 2011). Despite their structural similarity, different LhrCs have expression patterns that vary to some extent from one another (Mollerup et al., 2016). Cell envelope stress was shown to induce the expression of LhrC1-5 and Rli22 via the LisRK two-component system. Rli33-1 expression is upregulated in response to osmotic stress and during stationary growth phase in a Sigma B-dependent manner. Rli22 was found to be upregulated in bacteria inside the intestinal lumen of mice (Toledo-Arana et al., 2009). On the contrary, Rli22 is the only representative of the LhrC family which is not upregulated during growth inside macrophages (Mraheil et al., 2011). All seven ncRNAs were shown to be expressed after exposure to blood (Toledo-Arana et al., 2009; Table 1).

**FIGURE 2:**
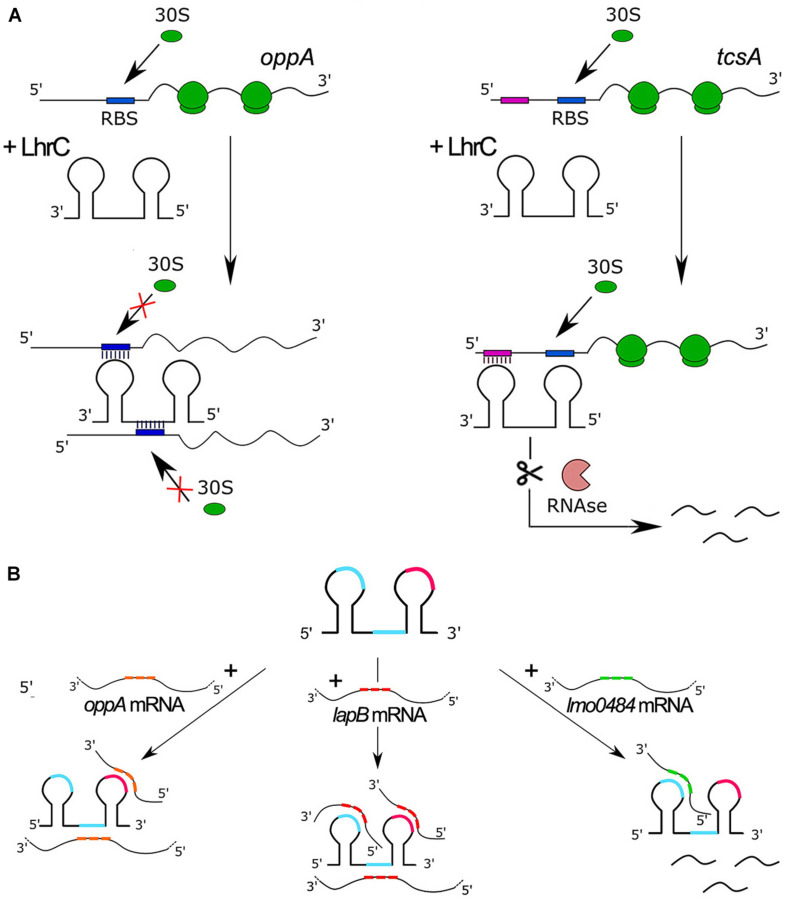
Features and regulatory mechanisms of ncRNAs from the LhrC family. **(A)** Model of LhrC regulation of *oppA* (left) and *tcsA* (right). The LhrC ncRNAs repress *oppA* expression by directly base paring to the RBS, leading to ribosome occlusion and repression of translation. In contrast, the LhrC ncRNAs repress *tcsA* expression by base pairing to a sequence far upstream of the RBS leading to degradation of *tcsA* mRNA without directly affecting translation. **(B)** Model of LhrCs and LhrC-target mRNA interactions. Each LhrC molecule possesses two different sites containing a UCCC motif located in loop A and the single-stranded region (blue). LhrC1-5 have an additional UCCC motif in the terminator loop (pink). CU-rich sequences are capable of binding to the AG-rich SD region of target mRNAs *oppA*, *lapB*, and *lmo0484.* One LhrC molecule may bind three *lapB* mRNAs, two *oppA* mRNAs or one *lmo0484* mRNA, and the target mRNAs show different binding preferences for the individual UCCC motifs.

To date, six targets for LhrCs have been identified. Five of these target genes encode surface proteins required for full virulence of *L. monocytogenes*. For three targets the mechanism of regulation has been studied in detail. The most important features and regulatory mechanisms of ncRNAs from the LhrC family are presented in Figure 2. The first target is *lapB* (*lmo1666*), which encodes a cell wall anchored adhesin. The second one is *oppA* (*lmo2349*) that encodes a substrate-binding protein of an oligopeptide transporter. The third target is *tcsA* (*lmo1388*), which encodes a CD4+ T cell-stimulating antigen. In two cases, the ncRNAs exert a negative effect on translation: for *lapB* and *oppA*, the LhrCs are known to act by direct base pairing to the ribosome binding site (RBS), leading to inhibition of translation followed by mRNA degradation (Sievers et al., 2014, 2015) (see Figure 2A). In the case of *tcsA*, the LhrC-binding site is located far upstream of the Shine-Dalgarno (SD) region in the 5′ UTR of the mRNA. Notably, the LhrCs act by promoting degradation of *tcsA* mRNA and do not affect the translation of this transcript (Sievers et al., 2015; Ross et al., 2019; Figure 2A). In addition to the three targets described above, the LhrCs are known to control the expression of genes involved in heme uptake and utilization: *lmo2186* and *lmo2185*, encoding the heme-binding proteins Hbp1 and Hbp2, respectively, and *lmo0484*, encoding a heme oxygenase-like protein. Using *in vitro* binding assays, it was shown that the LhrCs interact with mRNAs encoded from *lmo2186*, *lmo2185*, and *lmo0484*, and for *lmo0484* it was confirmed that the LhrC-binding site overlaps with the AG-rich SD region of the mRNA. Furthermore, LhrC1–5 down-regulate the expression of *lmo0484* at the posttranscriptional level in response to the cell wall-acting antibiotic cefuroxime through base pairing to the RBS, leading to inhibition of translation (dos Santos et al., 2018). While LhrC1-5 are known to interact with the chaperone Hfq, the interaction between LhrC and mRNAs has been shown so far to be Hfq-independent. A common feature of each LhrC family member is a structure that contains two stem loops (named stem loop A and terminator loop) joined by a single stranded stretch. Each LhrC family member contains two highly conserved UCCC motifs located in loop A and the single stranded stretch. LhrC1-5 contain an additional UCCC motif in the terminator loop. These motifs have been shown to be responsible for the interaction between these ncRNAs and their target mRNA sequences. Despite general similarity, the structure of the stem loops of Rli22 and Rli33-1 is slightly different from the structure of LhrC1-5. Interestingly, LhrC uses a different number of its UCCC motifs when pairing with different partners (see Figure 2B). For example, all three motifs in LhrC4 are capable of binding *lapB* mRNA, two are required for binding *oppA* mRNA, whereas only one is sufficient for efficient binding of *lmo0484* mRNA (Sievers et al., 2014, 2015; dos Santos et al., 2018). The unusually high number of binding sites of LhrC is considered to be a way of amplifying a weak input signal into a strong output response. Multiple binding sites may also accelerate the regulatory effect of LhrC, by binding multiple mRNAs at the same time. Additionally, different flanking regions adjacent to the UCCC motifs provide LhrC with a high degree of flexibility in terms of binding to SD regions of various target mRNAs.

### RliB

RliB displays five repeats of 29 nt spaced by 35–36 nt, strikingly resembling CRISPR (Clustered Regularly Interspaced Short Palindromic Repeats) elements present in many prokaryotes and archaea (Mandin et al., 2007). RliB is conserved at the same genomic locus in *L. monocytogenes* strains and also in other *Listeria* species (Mandin et al., 2007; Sesto et al., 2014). The deletion of *rliB* led to faster colonization of the livers of infected mice, indicating that RliB is involved in controlling virulence (Toledo-Arana et al., 2009; Table 1). A bioinformatic analysis was applied in an attempt to identify the targets of this ncRNA and this allowed prediction of three bicistronic transcripts as putative RliB targets, from which *lmo2104-lmo2105*, encoding the ferrous iron transport proteins FeoA and FeoB, respectively, was further analyzed (Mandin et al., 2007). The study revealed a weak complex formation ability between RliB and *lmo2104* and an increase of the *lmo2104-lmo2105* mRNA levels in *L. monocytogenes* as a consequence of overexpression of RliB, suggesting that an interaction of RliB with *lmo2104-lmo2105* mRNA may occur *in vivo* (Mandin et al., 2007). Further studies focused on the initially observed similarity of RliB to CRISPR elements and revealed that RliB is an atypical member of the CRISPR family, whose processing does not depend on *cas* (CRISPR-associated) genes (Sesto et al., 2014). Interestingly, an endogenously encoded polynucleotide phosphorylase (PNPase) with both 3′–5′ exoribonuclease and 3′ polymerase activities, has been identified as the enzyme responsible for processing of RliB-CRISPR into a 280 nt mature form. The PNPase-dependent processing of RliB-CRISPR is observed both in the *cas*-less *L. monocytogenes* strains and in those encoding a complete set of *cas* genes elsewhere in the genome. Functional studies revealed that RliB-CRISPR has DNA interference activity for which it requires the presence of both PNPase and the *cas* genes belonging to CRISPR-I (Sesto et al., 2014). These results indicate the involvement of RliB in the defense of *L. monocytogenes* against bacteriophage infection in strains carrying *cas* genes, however, the role of RliB in *cas*-less strains remains unknown. It is speculated that RliB-CRISPR’s *cas*-independent activity might rely on RNA interference that could be involved in controlling the formation of viral particles and lysis of the bacterial cell, transcription-dependent DNA targeting or gene expression silencing at the posttranscriptional level (Sesto et al., 2014). However, these hypotheses, as well as the discovery of the regulatory mechanism of RliB in *L. monocytogenes* virulence, require further research.

### RliI

The *rliI* gene is conserved in *L. innocua* and *Listeria ivanovii* species. The expression of *rliI* in *L. monocytogenes* does not change under conditions related to the infection process but it increases in the stationary phase of growth (Mandin et al., 2007; Toledo-Arana et al., 2009). Three putative bicistronic transcripts have been predicted as RliI targets, i.e., *lmo2660-lmo2659* encoding a transketolase and a ribulose-phosphate epimerase, *lmo1035-lmo1036* encoding a beta-glucoside transporter subunit of a PTS system and a beta-glucosidase, and *lmo2124-lmo2123* encoding components of a maltodextrin ABC transporter system. In each case, pairing of RliI with the 3′ ends of the target bicistronic mRNAs is anticipated (Mandin et al., 2007; Table 1). Among the anticipated mRNAs targets, binding of RliI with the *lmo1035-lmo1036* transcript has been examined and proven. Furthermore, it has been observed that overexpression of RliI in *L. monocytogenes* decreases the level of *lmo1035-lmo1036* mRNA, which suggests that RliI promotes the degradation of this transcript (Mandin et al., 2007). More recently, term-seq analysis, which maps the 3′-termini of RNA transcripts on a genome-wide scale, revealed that RliI is a putative conditional-terminator of the downstream gene *lmo2760* encoding an ABC transporter ATP-binding protein. This observation strongly suggests the role of RliI as a *cis* regulator (Dar et al., 2016). While the predicted function of the RliI targets suggests that this ncRNA is involved in controlling sugar metabolism and transport, the physiological role of RliI remains unknown.

### SbrE (Rli47)

The SbrE ncRNA is highly conserved among *L. monocytogenes* strains. In addition to *L. monocytogenes*, *sbrE* was also detected in the genomes of *L. innocua* and *Listeria welshimeri* (Mraheil et al., 2011). It was shown that SbrE affects the expression of the *lmo0636-lmo0637* operon (Mujahid et al., 2012). The Lmo0636 protein is predicted to be a DNA binding protein of the RrF2 family and Lmo0637 was annotated as an UbiE/COQ5 family methyltransferase. Additionally, in a mutant lacking *sbrE* a diminished level of Lmo2094, which is a metal ion binding class II aldolase/adducin domain protein, was observed. Studies of a *sbrE* deficient strain showed no significant effect of SbrE on growth in acid stress, salt stress, glucose-limiting conditions, or low temperature. Furthermore, the susceptibility of the *sbrE* mutant strain to infection with *Listeria* phages was comparable to the wild type strain (Mujahid et al., 2012). However, recent studies revealed that SbrE interacts with the SD region of the *ilvA* mRNA, which encodes threonine deaminase, an enzyme required for branched-chain amino acid biosynthesis (Marinho et al., 2019). Subsequent investigations revealed that in a mutant lacking the *sbrE* gene, both *ilvA* transcript levels and threonine deaminase activity were increased and the mutant also displayed a shorter growth lag in isoleucine-depleted growth media. These data indicate that SbrE acts to inhibit growth of *L. monocytogenes* under harsh conditions, through repression of isoleucine biosynthesis (Marinho et al., 2019). Furthermore, global transcriptional analyses revealed that SbrE is involved in modulation of amino acid metabolism and that the SbrE regulon largely overlaps with that of CodY, further establishing a possible role of Rli47 in the global regulation of metabolism during stress conditions (Marinho et al., 2019).

### Rli27

**FIGURE 3:**
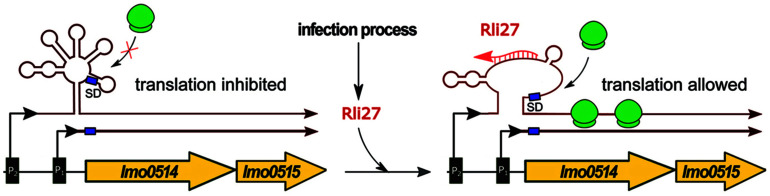
Regulatory mechanism of Rli27. In conditions not related to infection, the expression of *lmo0514-lmo0515* proceeds mainly from the constitutive P1 promoter as ribosomes cannot access the Shine-Dalgarno sequence (SD) within the long mRNA produced from the P2 promoter. During infection Rli27 is expressed and base-pairs with the 5′ UTR of the long transcript arising from the P2 promoter. The interaction between Rli27 and 5′ UTR of the long *lmo0514-lmo0515* mRNA allows ribosome binding and translation to proceed.

Rli27 is exclusive for the genus *Listeria*, with no orthologs found in other bacteria. Rli27 is responsible for the posttranscriptional regulation of *lmo0514*; the interaction region for Rli27 is located in the 5′ UTR of the target mRNA. Lmo0514 is an internalin-like protein with LPXTG motif, which is required for survival of *L. monocytogenes* in plasma (Quereda et al., 2016). It was shown that the abundance of Lmo0514 increases during infection of eukaryotic cells. The *lmo0514* gene was found to be transcribed from two promoters resulting in two mRNAs, containing a shorter and longer 5′ UTR sequence, respectively (Figure 3). The latter one, which contains the Rli27 interaction site, is detected exclusively in intracellular bacteria. Interaction between Rli27 and *lmo0514* long 5′ UTR mRNA has no effect on the transcript level of *lmo0514*, but results in increased translation. These observations suggest that the regulation is based on altering the secondary structure of the 5′ UTR that in turn leads to increased accessibility of the SD sequence (Quereda et al., 2014) (see Figure 3). Notably, the intracellular-specific translation of an alternative transcript, controlled by Rli27, is the only example described so far of positive regulation by *trans* ncRNAs in *L. monocytogenes*.

### Rli31

While Rli31 is highly abundant and expressed in all growth phases of *L. monocytogenes*, its transcription increases significantly during the infection of macrophage cells. Deletion of *rli31* results in decreased lysozyme resistance, decreased survival of *L. monocytogenes* in macrophages, and attenuation of virulence in insect and murine models of infection (Mraheil et al., 2011; Burke et al., 2014). Decreased mRNA levels of *pgdA* and *pbpX* (encoding peptidoglycan deacetylase and putative carboxypeptidase, respectively) were observed in the *rli31* mutant strain. However, Rli31 does not show any sequence complementarity to *pgdA* or *pbpX* transcripts, suggesting that Rli31 regulates expression of these genes in an indirect way (Burke et al., 2014). Rli31 was also proposed to function as the transcriptional attenuator of *lmo0559* encoding a Mg2^+^/Co2^+^ transporter, but this putative *cis*-regulation was not studied in detail (Dar et al., 2016). A further genetic screen for Rli31 target genes revealed that Rli31 binds the 5′ UTR of *spoVG* mRNA, as well as SpoVG protein (Table 1). However, despite its binding properties, Rli31 does not regulate SpoVG mRNA or protein abundance (Burke and Portnoy, 2016). Notably, SpoVG is a global regulator involved in lysozyme resistance, motility and virulence of *L. monocytogenes* and is itself able to bind various ncRNAs *in vitro* (Burke and Portnoy, 2016). Furthermore, inactivation of *rli31* and *spoVG* results in an opposite effect on lysozyme resistance and virulence, suggesting the existence of an antagonistic regulatory relationship between them. The molecular mechanism of Rli31 regulation and its link to SpoVG definitely requires further investigation.

### Rli32

Gene *rli32* is highly conserved in *L. monocytogenes*. The expression of Rli32 is stable in different conditions, including the intestinal lumen and whole human blood, but increases during infection of macrophage cells (Toledo-Arana et al., 2009; Mraheil et al., 2011). It has been shown that expression of Rli32 strongly depends on the transcriptional regulator of virulence VirR (Grubaugh et al., 2018). Furthermore, Rli32 holds the ability to bind to protein SpoVG, but the biological significance of this interaction remains unknown (Burke and Portnoy, 2016). More recently, a secRNome analysis of RNAs secreted by *L. monocytogenes* led to identification of Rli32 among ncRNAs that are secreted into the host cytoplasm following infection with *L. monocytogenes*, with strong β-interferon (IFN-β) inducing properties. The observed IFN-β expression triggered by Rli32 depends mainly on the presence of RIG-I (retinoic acid inducible gene I), and it was postulated that Rli32 is a ligand recognized by the RIG-I receptor (Frantz et al., 2019). Deletion of *rli32* results in decreased survival of *L. monocytogenes* in macrophages and increased resistance to the beta-lactam antibiotic cefuroxime. By contrast, the overexpression of Rli32 promotes intracellular bacterial growth and decreased resistance to cefuroxime. Additionally, *L. monocytogenes* overexpressing Rli32 is more resistant to H_2_O_2_ and exhibits increased catalase activity (Frantz et al., 2019). Comparative transcriptome analysis revealed that deletion of *rli32* led to the downregulation of ncRNA Rli60 and genes *lmo1627-lmo1633*, corresponding to the complete tryptophan operon, while the overexpression of Rli32 resulted in elevated expression of ncRNAs LhrC1-4, *lmo1958* and *lmo1960* encoding ferrichrome ABC transporter permease components, and operon *lmo2181-lmo2186* encoding heme-binding proteins Hbp1 and Hbp2, and components of a ferrichrome ABC transport system (Frantz et al., 2019). Especially intriguing seems the link between Rli32 and LhrC1-4, since inactivation of *rli32* and *lhrC1-4* results in an opposite effect on cefuroxime resistance. The molecular regulatory mechanism of Rli32 and its link to LhrC1-4 requires further investigation.

### Rli38

Rli38 is absent in non-pathogenic *L. innocua* and its expression is at least partially dependent on Sigma B (Toledo-Arana et al., 2009). Three putative mRNAs have been predicted as Rli38 targets, i.e., *lmo1956* (*fur*), *lmo0460*, and *lmo2752*. Notably, two of these genes encode proteins with roles in virulence: the transcriptional repressor Fur corresponds to the global iron uptake regulator and Lmo0460 is a membrane associated lipoprotein belonging to internalin family proteins. While the details of the interaction of Rli38 with its putative mRNA targets remain unknown, a functional analysis has shown that deletion of the *rli38* gene leads to attenuation of *L. monocytogenes* virulence in the murine model of infection, thus confirming the postulated importance of Rli38 in pathogenesis (Toledo-Arana et al., 2009).

### Rli50

Rli50 was first reported to be 176 nt in length (Toledo-Arana et al., 2009), but further studies revealed that the length of the Rli50 transcript is 306 nt (Mraheil et al., 2011). Moreover, it partially overlaps with Rli112 encoded from the opposite strand (Table 1). Rli50 shares homology with another ncRNA – Rli28 that is encoded from the region between *lmo0470* and *lmo0471* genes. Bioinformatic analysis showed that the chromosomal locus including *rli28* (*lmo0459–lmo0479*) has a different GC-content, which together with the presence of a IS3 family transposase gene (*lmo0464*) in this region led to speculation that horizontal gene transfer might be involved in chromosomal spreading of these regulatory RNAs (Mraheil et al., 2011). The Rli50 transcript level was slightly higher in extracellular *versus* intracellular conditions, but noteworthily, Rli50 is one of the most highly transcribed ncRNAs in *L. monocytogenes* under intracellular conditions. Deletion of the *rli50* gene resulted in decreased survival of *L. monocytogenes* in macrophage cells and led to attenuation of *L. monocytogenes* virulence in murine and insect models of infection. The attenuated virulence phenotype can be at least partially justified by the observed IFN-β induction in cells transfected with Rli50, which suggests the involvement of Rli50 in modulating the immune response during infection (Frantz et al., 2019). *In silico* studies showed that both Rli50 and Rli28 could pair with the mRNA of *lmo0549*, which shows similarity to an internalin-like gene. In addition, Rli50 is predicted to bind other ncRNAs such as Rli44, which suggests the existence of a regulatory network based on interaction between ncRNAs (Toledo-Arana et al., 2009). However, these putative interactions require experimental confirmation.

## *Cis* Regulatory ncRNAs

### Rli53

Initially, Rli53 was annotated as a conserved *cis* regulatory ncRNA located in the 5′ UTR of *lmo0919* (Table 1). However, it has also been hypothesized that Rli53 might function as a riboswitch, with an open state when *L. monocytogenes* resides in the intestinal lumen and a closed state in blood (Toledo-Arana et al., 2009). Recent term-seq studies revealed that Rli53 forms two alternative RNA structures that terminate or antiterminate the transcription of the downstream *lmo0919* gene, in response to the presence of the translation-inhibiting antibiotic lincomycin. In the absence of the antibiotic, transcription is terminated prematurely leading to the formation of a 207 nt Rli53 transcript. However, in the presence of the antibiotic, termination of Rli53 in the 5′ UTR of *lmo0919* is diminished, leading to increased transcription of *lmo0919* encoding an ABC transporter providing resistance to lincomycin (Dar et al., 2016). The predicted structure of Rli53 displays a conserved anti-antiterminator/antiterminator arrangement overlapping with a three-amino-acid ORF, which is translated in *L. monocytogenes.* Detailed functional studies revealed that the Rli53 regulatory mechanism relies on transcription attenuation mediated by lincomycin-inhibited ribosomes, which stall on the three-amino-acid ORF. This causes a shift of the riboregulator structure from a closed to an open state leading to induced expression of the full-length *lmo0919* mRNA that provides resistance to lincomycin (Dar et al., 2016).

### Rli59

Rli59 is a conserved *cis* regulatory ncRNA with a small ORF within its sequence (Toledo-Arana et al., 2009). Similar to Rli53, termination of Rli59 in the 5′ UTR of *lmo1652* is hampered by sub-lethal doses of translation-inhibiting antibiotics and leads to increased transcription of *lmo1652* encoding an ABC transporter with unknown function. However, the physiological effect of this regulation remains unknown, and furthermore regulation in this case is more permissive since Rli59 responds to different translation inhibiting antibiotics including lincomycin, erythromycin and chloramphenicol (Dar et al., 2016). While details concerning Rli59 regulation are missing, it is postulated that similarly to Rli53, Rli59 would control the level of *lmo1652* transcription via a mechanism of translation-coupled ribosome-mediated attenuation (Dar et al., 2016).

### Rli60

**FIGURE 4:**
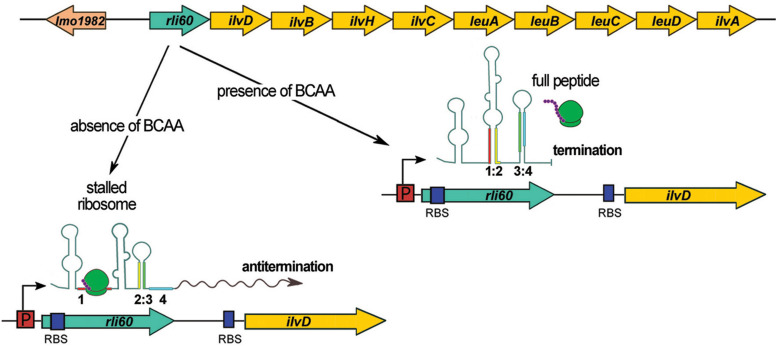
Regulatory mechanism of Rli60. In the upper panel, the schematic organization of the genome locus comprising *rli60* and BCAA biosynthesis operon (*ilv-leu*) is shown. In the lower panel, four regions of Rli60 are marked in colors (region 1: red, 2: yellow, 3: green, and 4: blue) illustrating the alternative structures that are formed. In the presence of BCAA, the leader peptide translating ribosome leads to formation of a terminator structure consisting of region 3 and 4. While transcription of *rli60* itself proceeds, the downstream genes of the *ilv-leu* operon are not transcribed. Upon a drop in BCAA levels the stalled ribosome (in region 1) leads to the formation of an alternative RNA structure between region 2 and 3, that antiterminates the transcription of the downstream genes of the BCAA biosynthesis operon.

Rli60 is encoded from the region upstream of *ilvD*, which is the first gene of the branched-chain amino acids (BCAA) biosynthesis operon *ilv-leu* (see Figure 4). Rli60 was predicted to function as a riboswitch with increased transcription in blood (Toledo-Arana et al., 2009) or as a ncRNA with a small ORF within its sequence (Mraheil et al., 2011). Other studies have suggested a role for Rli60 in stress adaptation, biofilm formation and virulence of *L. monocytogenes*, by a mechanism that is not known (Peng et al., 2016a, b). Recent studies revealed that *rli60* is co-transcribed with *ilvD* under BCAA limiting conditions, whereas under rich BCAA conditions a shorter transcript (∼200 nt) representing only Rli60 RNA is produced (Brenner et al., 2018; Figure 4). Of note, transcription of *ilvD* depends solely on transcription of *rli60* as *ilvD* does not possess a promoter on its own, meaning that transcription of *ilvD* is driven from the promoter located upstream of *rli60.* Detailed studies revealed that in low concentration of BCAA *rli60* is transcribed, forming two alternative RNA structures that terminate or antiterminate the transcription of the downstream *ilv-leu* genes. Transcription attenuation is dictated by a 13-amino-acid leader peptide rich in BCAA, which is translated ribosomally, implying that this mode of regulation corresponds to classical translation-coupled ribosome-mediated attenuation (Figure 4). Thus, Rli60 functions as a ribosome-mediated attenuator that regulates BCAA biosynthesis genes *in cis* and it is important for shutting down BCAA production even under BCAA depletion. This regulatory mechanism is crucial for virulence, as it ensures low level of the internal pools of BCAA, which is the signal for direct binding of the CodY regulator in the coding sequence of *prfA*. In turn, CodY activates the transcription of *prfA* and thereby stimulates the expression of virulence genes (Lobel et al., 2015; Brenner et al., 2018). Whether the 200 nt Rli60 ncRNA produced in BCAA rich condition functions as a *trans* acting regulator remains unknown. However, a recent study revealed that transfection of eukaryotic cells with Rli60 triggers moderate IFN-β induction (Frantz et al., 2019), suggesting that this ncRNA could have an additional role during infection that relies on modulating the immune response.

## lasRNAs (Excludons)

**FIGURE 5:**
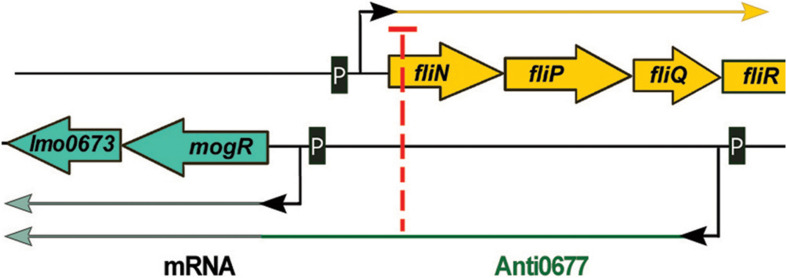
Regulatory mechanism of Anti0677 (excludon). The transcription of *anti0677* originates on the opposite strand to the coding sequence of three genes of the flagellum operon: *fliN*, *fliP*, and *fliQ*. The distal part of the excludon contains *mogR*, which encodes a transcriptional repressor of flagellum genes. The expression of *anti0677* leads to a decrease in the amount of *fliN*, *fliP*, and *fliQ* transcript due to base pairing and processing, and results in higher transcription of *mogR*, that in turn causes more efficient repression of transcription of the flagellum operon.

Anti0677 is an example of a unique class of lasRNA transcripts called excludons. These lasRNAs contain the mRNA sequence of a gene and an exceptionally long 5′ or 3′ UTR. The UTR region overlaps other genes transcribed on the opposite strand, affecting their expression. The transcription of *anti0677* originates on the opposite strand to the coding sequence of three genes of the flagellum operon: *fliN*, *fliP*, and *fliQ* and the distal part of the excludon contains the coding sequence of the *mogR* gene, which encodes a transcriptional repressor of flagellum genes (Table 1 and Figure 5). The *mogR* gene is transcribed from two promoters: transcription from the first, located just upstream of the start codon, generates a 1,200 nt transcript, whereas the second promoter is Sigma B dependent and drives transcription of a 2,900 nt Anti0677 excludon (Toledo-Arana et al., 2009). While the shorter *mogR* transcript is generated constitutively, *anti0677* was observed to be highly expressed during the stationary phase of growth. The expression of *anti0677* has a dual regulatory effect on the expression of the flagellum operon. First, it leads to higher transcription of *mogR*, that in turn causes more efficient repression of transcription of the flagellum operon. Second, it leads to a decrease in the amount of *fliN*, *fliP*, and *fliQ* transcript of flagellum operon due to base pairing and processing (Figure 5). Regarding a proposed action, the overexpression of *anti0677* was shown to impair motility (Toledo-Arana et al., 2009). While Anti0677 was the first described excludon of *L. monocytogenes*, further transcriptomic studies revealed the existence of additional excludons in this bacterium, i.e., Anti0605, Anti1846, and Anti0424 (Wurtzel et al., 2012). The transcription of *anti0605* inhibits expression of *lmo0605* encoding a MatE-family multidrug efflux pump and simultaneously leads to expression of *lmo0606* encoding a transcriptional regulator and two downstream genes (*lmo0607* and *lmo0608*) encoding an ABC-type multidrug transport system. Transcription of *anti1846* originates from the opposite strand relative to the coding sequence of *lmo1846* encoding an efflux pump from the MatE family. The distal part of the excludon contains the coding sequence of the downstream *lmo1845*, *lmo1844*, and *lmo1843* encoding xanthine-uracil permease, lipoprotein signal peptidase and ribosomal subunit synthase, respectively. It has been suggested that the *anti1846* might lead to expression of the permease while expression of the efflux pump is repressed. In the case of *anti0424*, it has been shown that this excludon includes genes involved in importing and metabolizing fructose (*lmo0425* and *lmo0428*) whereas its 5′ UTR overlaps, in the antisense orientation, with a glucose-specific permease (*lmo0424*). While functional studies are missing for these excludons, it is suggested that they may represent a common mechanism of linking regulation of physically adjacent genes that have opposing functions (Wurtzel et al., 2012).

## Riboswitches and Thermosensors

### PrfA Thermosensor

The PrfA thermosensor is a 127 nt riboregulator covering 115 nt of the 5′ UTR and 12 nt of the coding sequence of *prfA*, which encodes the master virulence regulator of *L. monocytogenes*. At environmental temperatures (30°C or below), the thermosensor element creates a stable hairpin structure which blocks the access of the ribosome to the SD sequence of *prfA* mRNA and therefore inhibits translation initiation. An increase of temperature to 37°C melts the stem-loop structure allowing the ribosome to access the SD sequence, resulting in translation of the *prfA* mRNA (Johansson et al., 2002; Table 1). The temperature-dependent control of *prfA* translation has a pivotal role in the virulence of *L. monocytogenes* as during infection of the host organism, the temperature rises to 37°C, opens the stem-loop and unmasks the translation initiation site. Consequently, PrfA is produced, which results in the transcription of the PrfA-dependent virulence genes.

### SreA Riboswitch

**FIGURE 6:**
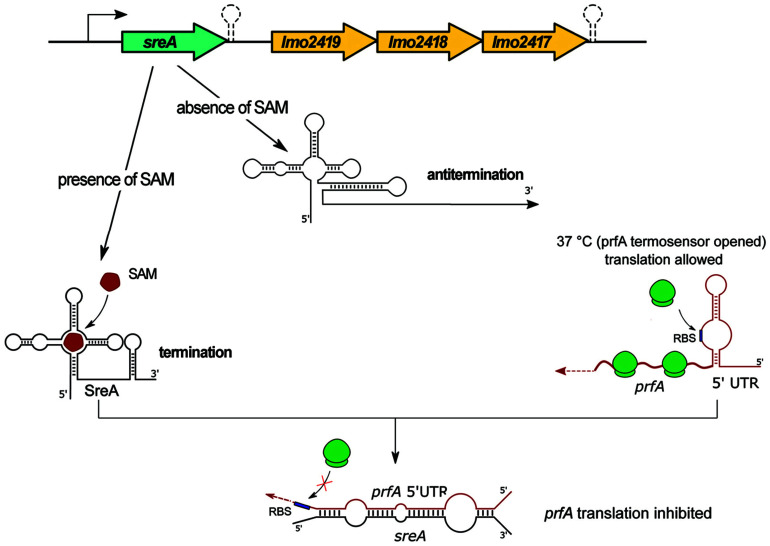
Regulatory mechanisms of the SreA riboswitch. The upper panel illustrates the schematic organization of the genome locus comprising the SAM riboswitch SreA and the *lmo2419-lmo2417* operon. In the absence of SAM the riboswitch element forms an antitermination structure that allows transcription of the downstream genes. Binding of SAM to the riboswitch alters its conformation, a terminator structure is formed, and downstream genes are not synthesized. The SreA small RNA, which is the product of a terminated riboswitch, base-pairs *in trans* with the 5′ UTR of *prfA* and blocks access of ribosomes to the SD sequence, which results in the inhibition of translation of *prfA*.

The SreA riboswitch, termed for SAM (*S*-adenosyl-methionine) riboswitch elements, is located upstream from, and in orientation consistent with, *lmo2419*, *lmo2418*, and *lmo2417* which encode proteins related to an ABC-transporter system potentially involved in methionine uptake (Figure 6 and Table 1). Interestingly, the SreA riboswitch exhibits dual function. First, SreA acts as a riboswitch to regulate *in cis* the expression of the *lmo2419-lmo2417* operon in a SAM-dependent manner. During growth in rich nutrient conditions, ensuring a high intracellular concentration of SAM, binding of the metabolite to the aptamer leads to premature termination of the operon transcription and formation of a truncated 229 nt transcript representing SreA alone. Contrary, during growth at low nutrient conditions, reflecting the absence of SAM, a full-length polycistronic transcript of around 2800 nt is produced (Loh et al., 2009). Therefore, in relation to regulation of the *lmo2419-lmo2417* operon, SreA exhibits a default structure and mechanism of action like other SAM riboswitches. Notably, in addition to the *in cis* activity of SreA on transcriptional regulation, the riboswitch can give rise to an ncRNA with *in trans* regulatory properties. More specifically, it was shown that SreA can base-pair, *in trans*, with the distal part of the 5′ UTR region of *prfA* mRNA. The interaction, which takes place approximately 80 nt upstream of the SD site of *prfA*, masks the SD sequence and impedes translation initiation (Loh et al., 2009). Thus, SreA acts as a dual riboregulator controlling the transcription of the downstream genes *in cis*, and furthermore, it acts *in trans* on distally located mRNAs, like *prfA* (Figure 6). Worth mentioning is that PrfA is a transcriptional activator of SreA expression. Therefore, SreA as a *trans* ncRNA constitutes part of a negative feedback loop on the expression of the main virulence regulator in *L. monocytogenes*. While the physiological effect of *sreA* inactivation was not examined, it is postulated that, according to the observed regulatory function on *prfA*, it is involved in virulence control. In agreement with this, SreA ncRNA is transcribed in the intestinal lumen and in blood (Toledo-Arana et al., 2009), therefore giving rise to *trans* regulatory activity of SreA in these conditions related to pathogenesis. On the other hand, a decreased level of the SreA ncRNA was observed during infection of macrophages (Mraheil et al., 2011), which suggests that SreA-mediated modulation of *prfA* expression changes at different stages of the infection process. Additionally, inactivation of *sreA* led to an increased level of *lmo2230* mRNA and decreased level of *lmo0049* mRNA. However, the regulatory mechanism of SreA on these mRNAs was not examined and therefore remains unknown. Notably, in addition to SreA, *L. monocytogenes* has six additional putative SAM riboswitches, from which SreB was shown to prevent *in trans* translation of *prfA* as well (Loh et al., 2009). SreB was also shown to restore the expression of *lmo2230*, but not that of *lmo0049* (Loh et al., 2009), suggesting that despite high similarity, the individual SAM riboswitches may differ in their ability to interact with mRNA targets.

### LysRS

The LysRS riboswitch, encoded between *lmo0798* and *lmo0799*, possesses a dual function. First, depending on the environmental conditions, the riboswitch acts as a terminator of transcription for the upstream gene *lmo0799*. The second regulatory mechanism of LysRS depends on the presence of lysine. Binding of the metabolite to the riboswitch leads to an alteration of its structure and results in transcription termination of the downstream gene *lmo0798* encoding a lysine transporter (Table 1). Notably, when lysine is absent, an anti-terminator structure is formed, which allows for transcription of the lysine transporter gene. Interestingly, in the absence of lysine a small transcript is generated, corresponding to LysRS alone (Toledo-Arana et al., 2009). The expression of *lysRS* proceeds from a Sigma B-dependent promoter, and its transcription increases in the presence of lysine and is strongly repressed during growth of *L. monocytogenes* in macrophages (Toledo-Arana et al., 2009; Mraheil et al., 2011). While the regulatory mechanism of LysRS is solved, its importance for *L. monocytogenes* physiology remains to be examined.

### Vitamin B_12_ Riboswitch of AspocR

**FIGURE 7:**
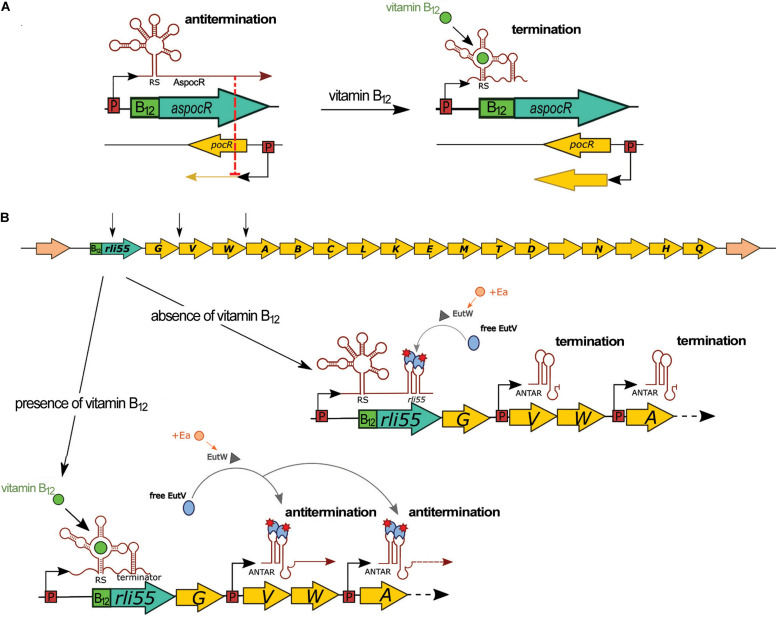
Regulatory mechanisms of vitamin B_12_ riboswitches. **(A)** Control of *pocR* by a B_12_ riboswitch and AspocR in the presence of propanediol. In the absence of vitamin B_12_, full-length AspocR is produced, which inhibits *pocR* expression. In the presence of vitamin B_12_, transcription of AspocR is terminated prematurely by the riboswitch, releasing transcription of *pocR*. **(B)** Control of the ethanolamine utilization (eut) operon by a B_12_ riboswitch and Rli55. In the upper panel, the schematic organization of the genome locus comprising Rli55 and *eut* genes is shown. EutW is a sensor kinase and EutV is an antiterminator of the two-component system EutWV. In the presence of ethanolamine (Ea), EutW activates EutV, which binds to ANTAR elements of Rli55 transcribed in absence of vitamin B_12_. When vitamin B_12_ is available, transcription of Rli55 terminates prematurely; therefore, EutV binds to ANTAR elements of *eutV* and *eutA* polycistronic transcripts and antiterminate the expression of the *eut* operon. Black arrows in the upper panel indicate the localization of ANTAR elements; RS, riboswitch.

A vitamin B_12_-dependent riboswitch is positioned between *lmo1149* and *lmo1150*, the latter encoding the transcriptional regulator PocR (Table 1). Initially, this riboswitch was annotated as ncRNA Rli39 and it was hypothesized to function as a riboswitch that terminates the transcription of *lmo1149* (Toledo-Arana et al., 2009). However, further research revealed that the riboswitch is transcribed as part of, and controls transcription of, an antisense RNA to *pocR* (AspocR) in a vitamin B_12_-dependent manner (Mellin et al., 2013; Figure 7A). Notably, AspocR encompasses as well the previously identified RliH and taking into account that no TSS was identified for RliH, this ncRNA is anticipated to be a processed fragment of AspocR (Mandin et al., 2007; Wurtzel et al., 2012; Mellin et al., 2013). Binding of vitamin B_12_ to the riboswitch leads to premature termination of *aspocR* transcription and the arising of a truncated 230 nt transcript. In the absence of vitamin B_12_, a full length AspocR transcript of 1,400 nt is produced, which inhibits *pocR* expression by an antisense mechanism (Figure 7A). PocR positively regulates expression of the *pdu* genes involved in propanediol utilization, as well as the *cob* genes responsible for biosynthesis of vitamin B_12_, in response to the presence of propanediol. These regulatory functions of PocR are linked, as propanediol catabolism requires vitamin B_12_ as a cofactor. The observed vitamin B_12_ riboswitch-dependent antisense regulation of *pocR* ensures that *pdu* genes are expressed only when both the substrate and the cofactor are available. It is ensured by only partial repression of *pocR* by AspocR in response to the presence of propanediol but in unavailability of vitamin B_12_. In such conditions, the arising level of PocR is sufficient to activate expression of the *cob* genes and synthesis of B_12_ cofactor, while propanediol catabolism genes are repressed. Therefore, the vitamin B_12_ riboswitch and AspocR serve as fine-tuning riboregulators integrating signals on propanediol and vitamin B_12_ availability (Mellin et al., 2013). While the regulatory mechanism of the riboswitch relies on modulation of transcription termination, the mechanistic details of AspocR regulation are elusive. AspocR inhibits *pocR* expression *in trans*, suggesting it acts through a direct interaction with *pocR* mRNA by a base-pairing mechanism rather than impeding the *pocR* transcription process. However, the possibility that AspocR may repress *pocR* expression at the level of transcription cannot be excluded. Furthermore, AspocR does not promote the degradation of *pocR* mRNA, which together with the higher level of PocR protein observed in the absence of AspocR *in vitro* suggests that AspocR interferes with translation initiation of *pocR* (Mellin et al., 2013). The expression of *aspocR* increases in the presence of propanediol and in the intestinal lumen, where propanediol is produced by commensal bacteria as a byproduct of the fermentation of rhamnose and fucose (Toledo-Arana et al., 2009; Mellin et al., 2013). While the effects of the vitamin B_12_ riboswitch or full length AspocR on pathogenesis of *L. monocytogenes* have not been examined, it is anticipated that the riboregulator plays a role in this process as propanediol catabolism is important for the pathogenesis of many intestinal pathogens (Mellin et al., 2013).

### Vitamin B_12_ Riboswitch of Rli55

The vitamin B_12_-dependent riboswitch is positioned upstream of Rli55 which is a ncRNA located in the close vicinity of the *eut* operon responsible for ethanolamine utilization (Toledo-Arana et al., 2009; Mellin et al., 2014; Figure 7B). Binding of vitamin B_12_ to the riboswitch leads to premature termination of Rli55 transcription, resulting in a short, 200 nt transcript, while in the absence of vitamin B_12_, a full-length transcript of around 450 nt is produced (Mellin et al., 2014). Further analysis revealed that full-length Rli55 contains a structural motif similar to ANTAR (amiR and nasR transcriptional antiterminator regulator) elements. This motif corresponds to a binding site for the EutV antiterminator of a two-component system, EutVW, which is responsible for the upregulation of the *eut* operon. The *eut* genes require both ethanolamine and vitamin B_12_ to be transcribed. In the presence of ethanolamine alone, EutV is bound and sequestered by the ANTAR element of Rli55. Conversely, in the presence of ethanolamine and vitamin B_12_, *rli55* transcription terminates prematurely downstream from the riboswitch, and therefore it is transcribed without an ANTAR element. This transcript cannot bind EutV, and thus allows EutV to bind ANTAR elements of *eut* mRNAs and antiterminate *eut* expression (Figure 7B; Mellin et al., 2014). As enzymes of the ethanolamine utilization pathway use vitamin B_12_ as a cofactor, Rli55 prevents expression of the *eut* locus in the absence of B_12_, thereby ensuring that the *eut* genes are expressed only in the presence of both substrate and cofactor. Deletion of the vitamin B_12_ riboswitch of Rli55 led to significantly reduced virulence; in contrast, deletion of the whole *rli55* sequence had no effect on virulence. Notably, Rli55 is the only ncRNA described so far in *L. monocytogenes* which regulates the expression of genes by the mechanism of the protein sequestration.

### CspA Thermosensor

CspA thermosensor is a 101 nt riboregulator located in the 5′ UTR of gene *cspA* encoding cold shock protein A (Table 1). At 37°C, the 5′ UTR creates a stable hairpin structure in the region of the SD sequence, making it unavailable for ribosome binding and therefore preventing translation initiation of *cspA*. A decrease of temperature to 30°C or below leads to the formation of a stable hairpin structure at the distal part of the 5′ UTR whereas the region containing the SD sequence becomes available for ribosome binding and thus translation of the *cspA* mRNA may proceed (Ignatov et al., 2020). The rearrangements of the thermosensor structure occur both *in vivo* and *in vitro*, indicating that a temperature change itself is sufficient to initiate conformational changes of the riboregulator. While the physiological role of thermosensor-driven control of *cspA* expression was not examined, it can be assumed that this regulatory mechanism is important for *L. monocytogenes* cold adaptation since CspA was shown to play an important role in cold stress tolerance (Schmid et al., 2009).

## Regulatory 5′ and 3′ UTRs

Recent studies revealed non-canonical posttranscriptional regulation, in which the 5′ and 3′ UTRs of *hly* mRNA are involved (Ignatov et al., 2020; Peterson et al., 2020; Table 1). The *hly* gene encodes listeriolysin O (LLO); a secreted pore-forming cytolysin that is a key virulence factor of *L. monocytogenes* (Cossart, 2011). LLO promotes rupture of the host phagosome membrane and therefore enables bacterial escape into the cytoplasm, where bacteria replicate and undergo cell-to-cell spreading. While the expression and activity of LLO is indispensable for *L. monocytogenes* virulence, it must be precisely regulated to ensure efficient escape of bacteria from a phagosome and to minimize cytotoxicity during growth inside the cytoplasm of infected cells. Recent work discovered that mRNA of *hly* forms an extensive secondary structure between the 5′ UTR comprising the RBS and a region encoding the PEST domain of LLO which is located near the N-terminus. The formation of this secondary mRNA structure is responsible for downregulation of LLO synthesis during bacterial growth (Peterson et al., 2020). Disruption of the interaction between the 5′ UTR*-*PEST sequence of *hly* mRNA did not change the level of *hly* mRNA but led to an increase of LLO. Further analysis revealed that this interaction is crucial for diminishing the cytotoxicity level during infection of host cells and therefore is important for *L. monocytogenes* virulence. Notably, 5′ UTR driven regulation of *hly* expression is observed only for growing bacteria. However, more details concerning the dependency of this regulatory mechanism on growth phase remain to be elucidated.

**FIGURE 8:**
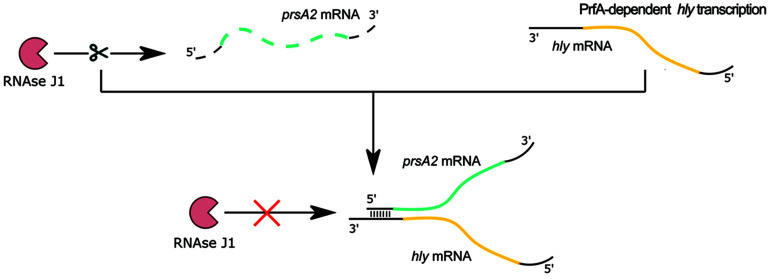
Regulatory mechanism of 3′ UTR of *hly* on *prsA2* mRNA. When the major virulence regulator PrfA is not active, transcription of the *hly* mRNA encoding the secreted virulence factor listeriolysin O does not proceed. In the absence of the *hly* transcript, the *prsA2* transcript is degraded by exoribonuclease RNase J1. After PrfA activation, *hly* transcription proceeds. The 3′ UTR of the arising *hly* mRNA base pairs with the distal 5′ end of the *prsA2* mRNA and protects it from RNase J1-mediated degradation.

The 3′ UTR of *hly* mRNA is also involved in posttranscriptional gene regulation. In studies devoted to the discovery of RNA-RNA and RNA-protein interactions, *prsA2* mRNA, encoding peptidyl-prolyl isomerase responsible for the folding of secreted proteins at the bacterial surface, was identified as the RNA target of posttranscriptional regulation by *hly* mRNA (Ignatov et al., 2020). Detailed studies revealed that the distal part of the 5′ UTR of *prsA2* mRNA directly interacts *in trans* with the 3′ UTR of full length *hly* mRNA. Disruption of the interaction between *hly* and *prsA2* mRNAs led to a reduction of *prsA2* mRNA level and protein abundance of PrsA2 but did not change *hly* mRNA level or the amount of LLO. The *hly-prsA2* interaction does not influence ribosome binding to the SD sequence of *prsA2* mRNA, but instead affects the stability of *prsA2* mRNA. Further analysis revealed that interaction with *hly* mRNA protects *prsA2* mRNA from degradation by RNase J1 (Figure 8), and that the *hly-*prsA2** interaction is important for *L. monocytogenes* virulence (Ignatov et al., 2020). Of note, the PrsA2 surface chaperone was shown to promote secretion and stability of LLO (Zemansky et al., 2009), therefore *hly*-driven regulation of *prsA2* constitutes a posttranscriptional mechanism ensuring efficient secretion and activity of the regulator. Notably, the *hly-*prsA2** interaction is the first described riboregulatory function of a 3′ UTR in *L. monocytogenes.*

## Concluding Remarks

The transcriptomic studies of recent years revealed the expression of a huge number of ncRNAs in *L. monocytogenes*. However, the biological functions and regulatory mechanisms of most ncRNAs remain unknown. Despite this fragmentary picture of the regulatory properties of the ncRNAs, recent research on RNA-mediated regulation in *L. monocytogenes* clearly points to ncRNAs being crucial contributors to virulence and stress adaptation. Strikingly, the vast majority of regulatory RNAs studied thus far are important for virulence. Moreover, through their regulatory functions at various stages of pathogenesis, these elements ensure successful infection by *L. monocytogenes*. In the intestinal lumen, effective growth of bacteria is ensured by vitamin B_12_ riboswitch-driven regulation (Mellin et al., 2014). In the blood, ncRNAs like the LhrCs and Rli27 contribute to resistance to heme toxicity and promote dissemination to deep lying organs (Quereda et al., 2016; dos Santos et al., 2018). Finally, in the intracellular phase of infection, efficient phagosome escape and low cytotoxicity inside the cytoplasm is ensured by riboregulatory elements involved in the control of LLO activity (Ignatov et al., 2020; Peterson et al., 2020). Importantly, multiple ncRNAs are known to regulate genes encoding cell envelope-associated proteins with virulence functions, such as *lapB*, *tcsA*, *hbp1, hbp2*, and *lmo0514* (Sievers et al., 2014; Quereda et al., 2014; Sievers et al., 2015; dos Santos et al., 2018; Ross et al., 2019). Furthermore, ncRNAs involved in regulation of amino acid biosynthesis genes, such as *ilvA* and *ilvD*, have been found to affect virulence (Brenner et al., 2018; Marinho et al., 2019). These findings indicate that riboregulators play important roles during infection by controlling *sensu stricto* virulence factors and by modulating the expression of genes involved in immune evasion, iron acquisition, and general metabolism. While the significance of iron transport and metabolism in the pathogenesis of *L. monocytogenes* is well known (Lechowicz and Krawczyk-Balska, 2015), recent research also points to a link between amino acid availability and virulence, as exemplified by increased virulence gene expression in response to a decrease in BCAA availability (Lobel et al., 2015). Therefore, adequate adjustments of different metabolic pathways is clearly important for establishing a successful infection, and ncRNAs appear to play important roles in these regulatory processes. Regulatory links between different metabolic pathways and the virulence program of *L. monocytogenes* can be achieved due to the versatility of ncRNAs. For example, the LhrC ncRNAs control the level of proteins belonging to different functional categories, such as amino acid and peptide transport, iron transport and metabolism, and surface proteins involved in virulence (Sievers et al., 2014; Sievers et al., 2015; dos Santos et al., 2018). Furthermore, SAM-dependent downregulation of *prfA* by SreA during growth in rich nutrient conditions also illustrates the important role of riboregulatory elements in the cross-coordination of virulence with metabolic pathways, and furthermore illustrates the complexity of riboregulation in this pathogen (Loh et al., 2009). This complexity is clearly manifested by the ability of regulatory RNAs to integrate complex metabolic stimuli, such as availability of different carbon sources and cofactors, into regulatory networks as exemplified by AspocR and Rli55 vitamin B_12_ riboswitches (Mellin et al., 2013, 2014). Presently, the complexity of regulation involving ncRNAs might be underestimated, as evidenced by the recently described Rli32-dependent changes in LhrC1-4 and Rli60 expression, which strongly suggests the existence of a regulatory network comprising multiple ncRNAs (Frantz et al., 2019). Intriguingly, multiple ncRNAs have been recently reported to modulate the innate immune response through interaction with host sensor RIG-I, suggesting that riboregulatory elements act to link and fine-tune the expression of both bacterial and host genes as a part of *L. monocytogenes*′ virulence strategies (Frantz et al., 2019).

Noteworthily, studies of riboregulation in *L. monocytogenes* have led to the definition of new concepts in prokaryotic gene regulation, such as the excludon, and disclosure of the versatility of riboswitches (Loh et al., 2009; Toledo-Arana et al., 2009; Wurtzel et al., 2012). However, further studies are required to explain the molecular basis and physiological role of unexplored post-transcriptional regulators. Such studies represent a demanding task, considering that riboregulators often represent fine-tuning, subtle modulation instead of all-or-nothing regulation. Notably, the regulatory function of most 5′ and 3′ UTRs is waiting to be revealed. Similarly, most excludons and their roles in adaptation to environmental conditions are still unknown. With a few exceptions, very little is known about RNA chaperones, such as Hfq, and other RNA binding proteins in *L. monocytogenes* (Jorgensen et al., 2020). So far, LhrA is the only example of an Hfq-dependent regulatory ncRNA in *L. monocytogenes*. Clearly, additional RNA binding proteins and their RNA partners remain to be uncovered in this bacterium. Furthermore, RNA-mediated regulation is triggered in response to changing environmental conditions, therefore the exploration of riboregulation demands linking to – and understanding of – the influence of specific environmental cues. Finally, an emerging theme in the field of riboregulation is the role of bacterial RNAs as virulence effectors modulating the expression of host genes during infection. In *L. monocytogenes*, the role of riboregulators in pathogen-host interactions most likely will continue being at the center of attention in the coming years.

## Author Contributions

AK-B and BK contributed to conception and design of the manuscript. AK-B wrote the first draft of the manuscript. AK-B, MŁ, and MB wrote sections of the manuscript. KŚ prepared figures. All authors contributed to manuscript revision, read, and approved the submitted version.

## Conflict of Interest

The authors declare that the research was conducted in the absence of any commercial or financial relationships that could be construed as a potential conflict of interest.

## References

[B1] Anast J. M. Schmitz-Esser S. (2020). The transcriptome of *Listeria monocytogenes* during co-cultivation with cheese rind bacteria suggests adaptation by induction of ethanolamine and 1, 2-propanediol catabolism pathway genes. *PLoS One* 15:e0233945. 10.1371/journal.pone.0233945 32701964 PMC7377500

[B2] Behrens S. Widder S. Mannala G. K. Qing X. Madhugiri R. Kefer N. (2014). Ultra deep sequencing of *Listeria monocytogenes* sRNA transcriptome revealed new antisense RNAs. *PLoS One* 9:e83979. 10.1371/journal.pone.0083979 24498259 PMC3911899

[B3] Brenner M. Lobel L. Borovok I. Sigal N. Herskovits A. A. (2018). Controlled branched-chain amino acids auxotrophy in *Listeria monocytogenes* allows isoleucine to serve as a host signal and virulence effector. *PLoS Genet.* 14:e1007283. 10.1371/journal.pgen.1007283 29529043 PMC5864092

[B4] Burke T. P. Portnoy D. A. (2016). SpoVG is a conserved RNA-binding protein that regulates *Listeria monocytogenes* lysozyme resistance, virulence, and swarming motility. *mBio* 7:e00240. 10.1128/mBio.00240-16 27048798 PMC4959528

[B5] Burke T. P. Loukitcheva A. Zemansky J. Wheeler R. Boneca I. G. Portnoy D. A. (2014). *Listeria monocytogenes* is resistant to lysozyme through the regulation, not the acquisition, of cell wall-modifying enzymes. *J. Bacteriol.* 196 3756–3767. 10.1128/JB.02053-14 25157076 PMC4248804

[B6] Christiansen J. K. Nielsen J. S. Ebersbach T. Valentin-Hansen P. Søgaard-Andersen L. Kallipolitis B. H. (2006). Identification of small Hfq-binding RNAs in *Listeria monocytogenes*. *RNA* 12 1383–1396. 10.1261/rna.49706 16682563 PMC1484441

[B7] Cossart P. (2011). Illuminating the landscape of host-pathogen interactions with the bacterium *Listeria monocytogenes*. *Proc. Natl. Acad. Sci. U.S.A.* 108 19484–19491. 10.1073/pnas.1112371108 22114192 PMC3241796

[B8] Cotter P. D. Emerson N. Gahan C. G. M. Hill C. (1999). Identification and disruption of lisRK, a genetic locus encoding a two-component signal transduction system involved in stress tolerance and virulence in *Listeria monocytogenes*. *J. Bacteriol.* 181 6840–6843. 10542190 10.1128/jb.181.21.6840-6843.1999 PMC94153

[B9] Dar D. Shamir M. Mellin J. R. Koutero M. Stern-Ginossar N. Cossart P. (2016). Term-seq reveals abundant ribo-regulation of antibiotics resistance in bacteria. *Science* 352:aad9822. 10.1126/science.aad9822 27120414 PMC5756622

[B10] Dorey A. Marinho C. Piveteau P. O’Byrne C. (2019). Role and regulation of the stress activated sigma factor sigma B (σB) in the saprophytic and host-associated life stages of *Listeria monocytogenes*. *Adv. Appl. Microbiol.* 106 1–48. 10.1016/bs.aambs.2018.11.001 30798801

[B11] dos Santos P. T. Menendez-Gil P. Sabharwal D. Christensen J.-H. Brunhede M. Z. Lillebæk E. M. S. (2018). The small regulatory RNAs LhrC1–5 contribute to the response of *Listeria monocytogenes* to heme toxicity. *Front. Microbiol.* 9:599. 10.3389/fmicb.2018.00599 29636750 PMC5880928

[B12] Ferreira V. Wiedmann M. Teixeira P. Stasiewicz M. J. (2014). *Listeria monocytogenes* persistence in food-associated environments: epidemiology, strain characteristics, and implications for public health. *J. Food Prot.* 77 150–170. 10.4315/0362-028X.JFP-13-150 24406014

[B13] Frantz R. Teubner L. Schultze T. La Pietra L. Müller C. Gwozdzinski K. (2019). The secRNome of *Listeria monocytogenes* harbors small noncoding RNAs that are potent inducers of Beta interferon. *mBio* 10:e01223-19. 10.1128/mBio.01223-19 31594810 PMC6786865

[B14] Grubaugh D. Regeimbal J. M. Ghosh P. Zhou Y. Lauer P. Dubensky T. W. (2018). The VirAB ABC transporter is required for VirR regulation of *Listeria monocytogenes* virulence and resistance to nisin. *Infect. Immun.* 86 e00901–17. 10.1128/IAI.00901-17 29263107 PMC5820956

[B15] Ignatov D. Vaitkevicius K. Durand S. Cahoon L. Sandberg S. S. Liu X. (2020). An mRNA-mRNA interaction couples expression of a virulence factor and its chaperone in *Listeria monocytogenes*. *Cell Rep.* 30 4027–4040.e7. 10.1016/j.celrep.2020.03.006 32209466 PMC8722363

[B16] Johansson J. Mandin P. Renzoni A. Chiaruttini C. Springer M. Cossart P. (2002). An RNA thermosensor controls expression of virulence genes in *Listeria monocytogenes*. *Cell* 110 551–561. 10.1016/s0092-8674(02)00905-4 12230973

[B17] Jorgensen M. G. Pettersen J. S. Kallipolitis B. H. (2020). sRNA-mediated control in bacteria: an increasing diversity of regulatory mechanisms. *Biochim. Biophys. Acta Gene Regul. Mech.* 1863:194504. 10.1016/j.bbagrm.2020.194504 32061884

[B18] Kallipolitis B. H. Ingmer H. Gahan C. G. Hill C. Søgaard-Andersen L. (2003). CesRK, a two-component signal transduction system in *Listeria monocytogenes*, responds to the presence of cell wall-acting antibiotics and affects beta-lactam resistance. *Antimicrob. Agents Chemother.* 47 3421–3429. 10.1128/aac.47.11.3421-3429.2003 14576097 PMC253798

[B19] Lebreton A. Cossart P. (2017). RNA- and protein-mediated control of *Listeria monocytogenes* virulence gene expression. *RNA Biol.* 14 460–470. 10.1080/15476286.2016.1189069 27217337 PMC5449094

[B20] Lechowicz J. Krawczyk-Balska A. (2015). An update on the transport and metabolism of iron in *Listeria monocytogenes*: the role of proteins involved in pathogenicity. *Biometals* 28 587–603. 10.1007/s10534-015-9849-5 25820385 PMC4481299

[B21] Liu D. Lawrence M. L. Ainsworth A. J. Austin F. W. (2007). Toward an improved laboratory definition of *Listeria monocytogenes* virulence. *Int. J. Food Microbiol.* 118 101–115. 10.1016/j.ijfoodmicro.2007.07.045 17727992

[B22] Lobel L. Sigal N. Borovok I. Belitsky B. R. Sonenshein A. L. Herskovits A. A. (2015). The metabolic regulator CodY links *Listeria monocytogenes* metabolism to virulence by directly activating the virulence regulatory gene *prfA*. *Mol. Microbiol.* 95 624–644. 10.1111/mmi.12890 25430920 PMC4329120

[B23] Loh E. Dussurget O. Gripenland J. Vaitkevicius K. Tiensuu T. Mandin P. (2009). A trans-acting riboswitch controls expression of the virulence regulator PrfA in *Listeria monocytogenes*. *Cell* 139 770–779. 10.1016/j.cell.2009.08.046 19914169

[B24] Mandin P. Fsihi H. Dussurget O. Vergassola M. Milohanic E. Toledo-Arana A. (2005). VirR, a response regulator critical for *Listeria monocytogenes* virulence. *Mol. Microbiol.* 57 1367–1380. 10.1111/j.1365-2958.2005.04776.x 16102006

[B25] Mandin P. Repoila F. Vergassola M. Geissmann T. Cossart P. (2007). Identification of new noncoding RNAs in *Listeria monocytogenes* and prediction of mRNA targets. *Nucleic Acids Res.* 35 962–974. 10.1093/nar/gkl1096 17259222 PMC1807966

[B26] Marinho C. M. Dos Santos P. T. Kallipolitis B. H. Johansson J. Ignatov D. Guerreiro D. N. (2019). The σB-dependent regulatory sRNA Rli47 represses isoleucine biosynthesis in *Listeria monocytogenes* through a direct interaction with the ilvA transcript. *RNA Biol.* 16 1424–1437. 10.1080/15476286.2019.1632776 31242083 PMC6779388

[B27] McLauchlin J. Mitchell R. T. Smerdon W. J. Jewell K. (2004). *Listeria monocytogenes* and listeriosis: a review of hazard characterisation for use in microbiological risk assessment of foods. *Int. J. Food Microbiol.* 92 15–33. 10.1016/S0168-1605(03)00326-X 15033265

[B28] Mellin J. R. Koutero M. Dar D. Nahori M.-A. Sorek R. Cossart P. (2014). Riboswitches. Sequestration of a two-component response regulator by a riboswitch-regulated noncoding RNA. *Science* 345 940–943. 10.1126/science.1255083 25146292

[B29] Mellin J. R. Tiensuu T. Bécavin C. Gouin E. Johansson J. Cossart P. (2013). A riboswitch-regulated antisense RNA in *Listeria monocytogenes*. *Proc. Natl. Acad. Sci. U.S.A.* 110 13132–13137. 10.1073/pnas.1304795110 23878253 PMC3740843

[B30] Mollerup M. S. Ross J. A. Helfer A.-C. Meistrup K. Romby P. Kallipolitis B. H. (2016). Two novel members of the LhrC family of small RNAs in *Listeria monocytogenes* with overlapping regulatory functions but distinctive expression profiles. *RNA Biol.* 13 895–915. 10.1080/15476286.2016.1208332 27400116 PMC5013991

[B31] Mraheil M. A. Billion A. Mohamed W. Mukherjee K. Kuenne C. Pischimarov J. (2011). The intracellular sRNA transcriptome of *Listeria monocytogenes* during growth in macrophages. *Nucleic Acids Res.* 39 4235–4248. 10.1093/nar/gkr033 21278422 PMC3105390

[B32] Mujahid S. Bergholz T. M. Oliver H. F. Boor K. J. Wiedmann M. (2012). Exploration of the role of the non-coding RNA SbrE in *L. monocytogenes* stress response. *Int. J. Mol. Sci.* 14 378–393. 10.3390/ijms14010378 23263668 PMC3565269

[B33] Nielsen J. S. Larsen M. H. Lillebæk E. M. S. Bergholz T. M. Christiansen M. H. G. Boor K. J. (2011). A small RNA controls expression of the chitinase ChiA in *Listeria monocytogenes*. *PLoS One* 6:e19019. 10.1371/journal.pone.0019019 21533114 PMC3078929

[B34] Nielsen J. S. Lei L. K. Ebersbach T. Olsen A. S. Klitgaard J. K. Valentin-Hansen P. (2010). Defining a role for Hfq in Gram-positive bacteria: evidence for Hfq-dependent antisense regulation in *Listeria monocytogenes*. *Nucleic Acids Res.* 38 907–919. 10.1093/nar/gkp1081 19942685 PMC2817478

[B35] Nielsen J. S. Olsen A. S. Bonde M. Valentin-Hansen P. Kallipolitis B. H. (2008). Identification of a σB-dependent small noncoding RNA in *Listeria monocytogenes*. *J. Bacteriol.* 190 6264–6270. 10.1128/JB.00740-08 18621897 PMC2546787

[B36] Oliver H. F. Orsi R. H. Ponnala L. Keich U. Wang W. Sun Q. (2009). Deep RNA sequencing of *L. monocytogenes* reveals overlapping and extensive stationary phase and sigma B-dependent transcriptomes, including multiple highly transcribed noncoding RNAs. *BMC Genomics* 10:641. 10.1186/1471-2164-10-641 20042087 PMC2813243

[B37] Peng Y.-L. Meng Q.-L. Qiao J. Xie K. Chen C. Liu T.-L. (2016a). The regulatory roles of ncRNA Rli60 in adaptability of *Listeria monocytogenes* to environmental stress and biofilm formation. *Curr. Microbiol.* 73 77–83. 10.1007/s00284-016-1028-6 27032404

[B38] Peng Y.-L. Meng Q.-L. Qiao J. Xie K. Chen C. Liu T.-L. (2016b). The roles of noncoding RNA Rli60 in regulating the virulence of *Listeria monocytogenes*. *J. Microbiol. Immunol. Infect.* 49 502–508. 10.1016/j.jmii.2014.08.017 25442865

[B39] Peterson B. N. Portman J. L. Feng Y. Wang J. Portnoy D. A. (2020). Secondary structure of the mRNA encoding listeriolysin O is essential to establish the replicative niche of *L. monocytogenes*. *Proc. Natl. Acad. Sci. U.S.A.* 117 23774–23781. 10.1073/pnas.2004129117 32878997 PMC7519334

[B40] Quereda J. J. García-Del Portillo F. Pucciarelli M. G. (2016). *Listeria monocytogenes* remodels the cell surface in the blood-stage. *Environ. Microbiol. Rep.* 8 641–648. 10.1111/1758-2229.12416 27085096

[B41] Quereda J. J. Ortega ÁD. Pucciarelli M. G. García-del Portillo F. (2014). The *Listeria* small RNA Rli27 regulates a cell wall protein inside eukaryotic cells by targeting a long 5′-UTR variant. *PLoS Genet.* 10:e1004765. 10.1371/journal.pgen.1004765 25356775 PMC4214639

[B42] Reniere M. L. Whiteley A. T. Portnoy D. A. (2016). An in vivo selection identifies *Listeria monocytogenes* genes required to sense the intracellular environment and activate virulence factor expression. *PLoS Pathog.* 12:e1005741. 10.1371/journal.ppat.1005741 27414028 PMC4945081

[B43] Renzoni A. Cossart P. Dramsi S. (1999). PrfA, the transcriptional activator of virulence genes, is upregulated during interaction of *Listeria monocytogenes* with mammalian cells and in eukaryotic cell extracts. *Mol. Microbiol.* 34 552–561. 10.1046/j.1365-2958.1999.01621.x 10564496

[B44] Ross J. A. Thorsing M. Lillebæk E. M. S. Teixeira Dos Santos P. Kallipolitis B. H. (2019). The LhrCsRNAs control expression of T cell-stimulating antigen TcsA in *Listeria monocytogenes* by decreasing tcsA mRNA stability. *RNA Biol.* 16 270–281. 10.1080/15476286.2019.1572423 30706751 PMC6380316

[B45] Schmid B. Klumpp J. Raimann E. Loessner M. J. Stephan R. Tasara T. (2009). Role of cold shock proteins in growth of *Listeria monocytogenes* under cold and osmotic stress conditions. *Appl. Environ. Microbiol.* 75 1621–1627. 10.1128/AEM.02154-08 19151183 PMC2655451

[B46] Schultze T. Izar B. Qing X. Mannala G. K. Hain T. (2014). Current status of antisense RNA-mediated gene regulation in *Listeria monocytogenes*. *Front. Cell Infect. Microbiol.* 4:135. 10.3389/fcimb.2014.00135 25325017 PMC4179725

[B47] Sesto N. Touchon M. Andrade J. M. Kondo J. Rocha E. P. C. Arraiano C. M. (2014). A PNPase dependent CRISPR system in *Listeria*. *PLoS Genet.* 10:e1004065. 10.1371/journal.pgen.1004065 24415952 PMC3886909

[B48] Sievers S. Lund A. Menendez-Gil P. Nielsen A. Storm Mollerup M. Lambert Nielsen S. (2015). The multicopy sRNA LhrC controls expression of the oligopeptide-binding protein OppA in *Listeria monocytogenes*. *RNA Biol.* 12 985–997. 10.1080/15476286.2015.1071011 26176322 PMC4615310

[B49] Sievers S. SternkopfLillebæk E. M. Jacobsen K. Lund A. Mollerup M. S. Nielsen P. K. (2014). A multicopy sRNA of *Listeria monocytogenes* regulates expression of the virulence adhesinLapB. *Nucleic Acids Res.* 42 9383–9398. 10.1093/nar/gku630 25034691 PMC4132741

[B50] Storz G. Vogel J. Wassarman K. M. (2011). Regulation by small RNAs in bacteria: expanding frontiers. *Mol. Cell* 43 880–891. 10.1016/j.molcel.2011.08.022 21925377 PMC3176440

[B51] Toledo-Arana A. Dussurget O. Nikitas G. Sesto N. Guet-Revillet H. Balestrino D. (2009). The *Listeria* transcriptional landscape from saprophytism to virulence. *Nature* 459 950–956. 10.1038/nature08080 19448609

[B52] Waters L. S. Storz G. (2009). Regulatory RNAs in bacteria. *Cell* 136 615–628. 10.1016/j.cell.2009.01.043 19239884 PMC3132550

[B53] Wehner S. Mannala G. K. Qing X. Madhugiri R. Chakraborty T. Mraheil M. A. (2014). Detection of very long antisense transcripts by whole transcriptome RNA-Seq analysis of *Listeria monocytogenes* by semiconductor sequencing technology. *PLoS One* 9:e108639. 10.1371/journal.pone.0108639 25286309 PMC4186813

[B54] Wurtzel O. Sesto N. Mellin J. R. Karunker I. Edelheit S. Bécavin C. (2012). Comparative transcriptomics of pathogenic and non-pathogenic *Listeria* species. *Mol. Syst. Biol.* 8:583. 10.1038/msb.2012.11 22617957 PMC3377988

[B55] Zemansky J. Kline B. C. Woodward J. J. Leber J. H. Marquis H. Portnoy D. A. (2009). Development of a mariner-based transposon and identification of *Listeria monocytogenes* determinants, including the peptidyl-prolyl isomerase PrsA2, that contribute to its hemolytic phenotype. *J. Bacteriol.* 191 3950–3964. 10.1128/JB.00016-09 19376879 PMC2698408

